# Erythrocyte Phospholipid and Polyunsaturated Fatty Acid Composition in Diabetic Retinopathy

**DOI:** 10.1371/journal.pone.0106912

**Published:** 2014-09-04

**Authors:** Philippe Koehrer, Sarah Saab, Olivier Berdeaux, Rodica Isaïco, Stéphane Grégoire, Stéphanie Cabaret, Alain M. Bron, Catherine P. Creuzot-Garcher, Lionel Bretillon, Niyazi Acar

**Affiliations:** 1 Department of Ophthalmology, University Hospital, Dijon, France; 2 INRA, UMR1324 Centre des Sciences du Goût et de l’Alimentation, Dijon, France; 3 CNRS, UMR6265 Centre des Sciences du Goût et de l’Alimentation, Dijon, France; 4 Université de Bourgogne, UMR Centre des Sciences du Goût et de l’Alimentation, Dijon, France; Queen’s University Belfast, United Kingdom

## Abstract

**Background:**

Long chain polyunsaturated fatty acids (LCPUFAs) including docosahexaenoic acid and arachidonic acid are suspected to play a key role in the pathogenesis of diabetes. LCPUFAs are known to be preferentially concentrated in specific phospholipids termed as plasmalogens. This study was aimed to highlight potential changes in the metabolism of phospholipids, and particularly plasmalogens, and LCPUFAs at various stages of diabetic retinopathy in humans.

**Methodology and Principal Findings:**

We performed lipidomic analyses on red blood cell membranes from controls and mainly type 2 diabetes mellitus patients with or without retinopathy. The fatty acid composition of erythrocytes was determined by gas chromatography and the phospholipid structure was determined by liquid chromatography equipped with an electrospray ionisation source and coupled with a tandem mass spectrometer (LC-ESI-MS/MS). A significant decrease in levels of docosahexaenoic acid and arachidonic acid in erythrocytes of diabetic patients with or without retinopathy was observed. The origin of this decrease was a loss of phosphatidyl-ethanolamine phospholipids esterified with these LCPUFAs. In diabetic patients without retinopathy, this change was balanced by an increase in the levels of several phosphatidyl-choline species. No influence of diabetes nor of diabetic retinopathy was observed on the concentrations of plasmalogen-type phospholipids.

**Conclusions and Significance:**

Diabetes and diabetic retinopathy were associated with a reduction of erythrocyte LCPUFAs in phosphatidyl-ethanolamines. The increase of the amounts of phosphatidyl-choline species in erythrocytes of diabetic patients without diabetic retinopathy might be a compensatory mechanism for the loss of LC-PUFA-rich phosphatidyl-ethanolamines.

## Introduction

Diabetic retinopathy (DR) is a microvascular complication of diabetes representing the first cause of blindness in the US and Europe before the age of 50 [1]. The activation of biochemical pathways by hyperglycemia, such as protein kinase C (PKC), aldolase-reductase and/or advanced glycation endproducts pathways, and oxidative pathways leads to retinal ischemia by extensive capillary abnormalities [2], [3]. Among them, capillary occlusions lead to retinal ischemia and pre-retinal neovascularization through Vascular Endothelial Growth Factor (VEGF) production [4]. Neovascular processes are further responsible for vision threatening complications such as tractional retinal detachment or neovascular glaucoma.

Long chain polyunsaturated fatty acids (LCPUFAs) including docosahexaenoic acid (DHA, C22∶6n-3) and arachidonic acid (AA, C20∶4n-6) are suspected to play key functions in the pathogenesis of diabetes as glucose and lipid metabolisms are closely related [5]. Indeed, a shift from unsaturated to saturated fatty acids in cell membranes, as observed at preclinical stages of diabetes, is suspected to reduce erythrocytes deformability and subsequently oxygen supply to tissues, thus promoting microvascular complications of diabetes [6]. Modifications in lipid composition is also suspected to impact glucose effectiveness and insulin sensitivity, as shown by a recent meta-analysis on n-3 LCPUFA bioavailability and insulin sensitivity [7]. LCPUFAs from the n-3 family have been shown to inhibit many cellular and biochemical processes involved in the pathophysiology of DR, namely the PKC, aldolase reductase, and advanced glycation endproducts pathways, as well as the expression of VEGF, the loss of pericytes, and platelet aggregation [5].

As for the brain, the retina is characterized by its high content in LCPUFAs carried by phospholipids [8]–[10]. These phospholipids consist of a glycerol backbone connected to two fatty acid radicals at the *sn*-1 and *sn*-2 positions, and to a polar head group at the *sn*-3 position of glycerol. Depending on the nature of the polar head-group, the two main classes of phospholipids are phosphatidyl-ethanolamine (PE) and phosphatidyl-choline (PC). As for other tissues or cell types, retinal phospholipids also consist of particular phospholipids called plasmalogens, which have a fatty alcohol radical and a vinyl-ether bond at *sn*-1 instead of a fatty acid radical [11]. As with conventional phospholipids, plasmalogens are classified according to their *sn*-3 position, the most abundant plasmalogens being plasmenyl-ethanolamine (PlsE) and plasmenyl-choline (PlsC). Because of the preferential esterification of LCPUFAs at their *sn*-2 position [12]–[14], plasmalogens are considered as reservoirs of LCPUFAs in membranes.

Several studies have shown modifications of the fatty acid but also of the phospholipid content of cell membranes in human diabetic patients and in animal models of diabetes [15]–[18]. However, the specific contribution of plasmalogens was not investigated so far, especially in diabetic retina where LCPUFAs were shown to prevent the retinal vascular damage caused by diabetes [19]. The aim of our work was to highlight potential changes in the metabolism of LCPUFAs in relationship with their phospholipid origin in DR. For that purpose, we performed lipidomic analyses of phospholipids in erythrocyte membranes from diabetic patients with or without retinopathy.

## Materials and Methods

### Ethics Statement

Collection of the samples from subjects was conducted in accordance with the guidelines of the Declaration of Helsinki. A written consent was obtained and the protocol was accepted by the local ethics committee (CPP Est I, Faculty of Medicine, Dijon, France).

### Selection of the human subjects and sample collection

Control subjects and type 1 or 2 diabetes mellitus patients were recruited in the Department of Ophthalmology, University Hospital, Dijon, France between June 2011 and April 2012. Histories of panretinal photocoagulation, laser macular grid or intravitreal injections of anti-VEGF or corticosteroids were considered as exclusion criteria. No subject treated with statin or any hypolipidemic drug was included in the study. The staging of DR was determined after pupillary dilation on fundus photographs (Visucam Pro NM retinal camera, Zeiss Meditec, Le Pecq, France) of 8 peripheral retina fields by using the classification of the Early Treatment Diabetic Retinopathy Study (ETDRS) [20]. Absence of diabetes mellitus among control subjects was confirmed by glucose fasting test (data not shown). A blood sample was collected at the time of the ophthalmologic examination in heparinized tubes by venipuncture. Triglycerides and total-, LDL- and HDL-cholesterol were quantified by standard automatic analyzers at the Clinical Chemistry Department of the University Hospital (Dijon, France). Red blood cells were separated from plasma by centrifugation at 3000 rpm for 10 min at +4°C. All samples were immediately stored at –80°C until further analyses.

### Lipid analyses

#### Lipid extraction from erythrocytes

Lipids were extracted from erythrocytes according to Moilanen and Nikkari [21]. Phospholipids were purified from total lipid extracts using silica cartridges as previously described [9], [22], [23]. Phospholipid extracts were stored under inert gas until further analyses.

#### Fatty acid analysis by gas chromatography

Total phospholipids from red blood cells were transmethylated using boron trifluoride in methanol according to Morrison and Smith [24]. Fatty acid methyl esters (FAMEs) and dimethylacetals (DMAs) were subsequently extracted with hexane and analyzed on a Hewlett Packard Model 5890 gas chromatograph (Palo Alto, CA, USA) using a CPSIL-88 column (100****m×0.25****mm i.d., film thickness 0.20** µ**m; Varian, Les Ulis, France) equipped with a flame ionization detector. Hydrogen was used as carrier gas (inlet pressure 210 kPa). The oven temperature was held at 60°C for 5****min, increased to 165°C at 15°C/min and held for 1****min, and then to 225°C at 2°C/min and finally held at 225°C for 17****min. The injector and the detector were maintained at 250°C. FAMEs and DMAs were identified by comparison with commercial and synthetic standards. The data were processed using the EZChrom Elite software (Agilent Technologies, Massy, France) and reported as a percentage of the total fatty acids.

#### Structural analysis of phospholipids by LC-ESI-MS

Prior to LC-ESI-MS analyses, the phosphorus content of total phospholipid extracts was determined according to Bartlett and Lewis [25]. After the internal standards dimyristoyl-sn-glycerol-3-phosphatidylethanolamine (PE14**∶**0/14**∶**0) and dimyristoyl-sn-glycerol-3-phosphatidylcholine (PC14**∶**0/14**∶**0) in chloroform/methanol (1**∶**1, v/v) were added, samples were stored at **−**80°C under argon atmosphere.

The structural analysis of phospholipids was performed according to previously described procedures [9], [26]. Briefly, liquid chromatography separation was performed using a Hypersil Gold Silica Column (150 mm×2.1 mm i.d.×3 µm, ThermoFinnigan, San Jose, CA, USA) and a mobile phase consisting of: (A) hexane/propan-2-ol/chloroform/water (44/43.5/10.5/2, v/v/v/v) containing 12.5 mM of ammonium formate, and (B) hexane/propan-2-ol/chloroform/water (34/49/10.5/6.5, v/v/v/v) containing 12.5 mM of ammonium formate. The solvent-gradient system was as follows: 0 min A/B (%) 100/0, 10.5–24 min A/B (%) 22/78, 26.5–45 min A/B (%) 0/100% and 46–60 min A/B (%) 100/0. The flow rate was of 300 µL.min^−1^.

Mass spectrometry was performed using a ThermoFinnigan TSQ Quantum triple quadrupole mass spectrometer (ThermoFinnigan, San Jose, CA, USA) equipped with a standard electrospray ionisation source. Nitrogen was used as sheath and auxiliary gas. The electrospray ionisation spray voltages were of 3 kV and −4.5 kV in negative and positive ion modes, respectively. Vaporiser temperature was of 150°C, sheath gas N_2_ pressure 45 (arbitrary unit), auxiliary gas pressure 45 (arbitrary unit), ion sweep gas pressure 5, ion transfer capillary temperature 300°C, skimmer offset 5 V and multiplier gain 300,000.

PE and PC species were manually identified with the parent mass information and their characteristic fragment ions in the CID spectrum using a local database [9], [26].

For all calculations, the ratio of peak area of each PC and PE specie to the peak area of the internal standards (PC14∶0/14∶0 or PE14∶0/14∶0, respectively) were used. Since the use of the neutral loss of 141 Da is problematic in the quantification of PlsE [27], quantification was based on multiple reaction monitoring (MRM) of one parent/fragment transition for each selected plasmalogen whereas PE14∶0/14∶0 was considered as internal standard. The standard curves were then plotted as ratio height of the respective PlsE specie/height of PE14∶0/14∶0 versus the concentration. The data were processed using the Xcalibur software (ThermoFinnigan). Corrections were applied to data for isotopic overlap.

### Statistical analyses

Statistical analyses were performed using the Statistical Analysis System (SAS Institute, Cary, NC, USA). Data with a normal distribution are expressed as mean ± standard deviation (SD) or mean ± standard error of the mean (SEM) whereas those having a non-normal distribution are as median and interquartile range [IQR]. The Kruskal-Wallis test was used to compare different groups for continuous variables. The chi-square test was used to compare dichotomous variables between the different groups. A post-test was done using the post-hoc Dunn’s test. The tests were two-tailed and *P* values lower than 0.05 were considered as statistically significant.

## Results

Patient characteristics are displayed in **Table 1** **.** We included 102 individuals, 53 men and 49 females. These were 18 control subjects, 14 diabetic patients without DR, 12 mild non-proliferative DR patients, 12 moderate non-proliferative DR patients, 22 severe non-proliferative DR patients, and 24 proliferative DR patients. Our population was almost exclusively composed of type 2 diabetic patients. The number of type 1 diabetic patient in each group was very low and the ratio of type 1/type 2 diabetes was not statistically different among groups (P = 0.27). There was no significant difference for age (P = 0.58) and gender (P = 0.91) between groups. HBA_1c_ level did not differ between diabetic patients subgroups (P = 0.44). All patients displayed normal values of plasma triglycerides, total-, LDL- and HDL-cholesterol. No difference was observed between the study groups on plasma lipid parameters. There was no significant difference for the rate of diabetic nephropathy between the groups of diabetic patients (P = 0.42) or for macrovascular complications of diabetes such as coronaropathy, peripheral arteriosclerosis (P = 0.84 and P = 0.59, respectively).

**Table 1:** Characteristics of the patients included in the study.

	Controls	Diabeticsubjects	Patients with diabetic retinopathy				total	*P* a
			mild	moderate	severe	proliferative		
**Gender (F/M)**	9/9	7/7	5/7	8/4	14/8	10/14	53/49	0.58
**Age (yr)** *mean ± SD*	64.5±11.8	64.7±14.5	58.2±20.2	64.8±14.4	65.8±13.1	61.5±13.1	62.6±14.9	0.91
**n = **	18	14	12	12	22	24	102	-
**Type I diabetes** **(n)/Type II diabetes (n)**	-	1/13	1/11	1/11	3/19	7/17	13/71	0.27
**Diabetic** **nephropathy (n/%)**	-	1/7.1	4/25.0	3/25.0	8/35.0	7/29.2	23/27.4	0.42
**Coronaropathy (n/%)**	-	3/21.4	3/25.0	5/41.6	6/28.0	7/29.2	24/28.6	0.84
**Peripheral** **arteriosclerosis (n/%)**	-	0/0	2/16.7	2/16.7	4/18.2	4/16.7	12/14.2	0.59
**HBA_1c_**		7.7±2.2	8.0±0.9	7.7±1.0	8.3±1.7	8.2±1.1		
**%**	-						-	0.44
**(mmol/mol)**		(60.9±24.7)	(64.6±10.4)	(61.4±11.4)	(67.1±19.0)	(66.5±13.0)		
*mean ± SD*								
**Total cholesterol**								
**(mmol/L)**	4.36±0.20	5.05±0.25	4.81±0.37	4.57±0.61	4.62±0.38	4.85±0.38	-	0.40
*mean ± SEM*								
**LDL cholesterol**								
**(mmol/L)**	2.46±0.22	2.51±0.28	2.57±0.32	2.40±0.44	2.28±0.38	2.63±0.39	-	0.98
*mean ± SEM*								
**HDL cholesterol**								
**(mmol/L)**	1.57±0.10	1.56±0.24	1.40±0.20	1.37±0.21	1.55±0.25	1.52±0.22	-	0.64
*mean ± SEM*								
**Triglycerides**								
**(mmol/L)**	1.14±0.08	1.39±0.40	1.70±0.39	1.67±0.41	1.50±0.17	1.55±0.20	-	0.11
*mean ± SEM*								

a Based on Chi-square test for gender, type of diabetes and micro- and macro-vascular complications, and Kruskall-Wallis test for age, HBA_1c_, total cholesterol, LDL cholesterol, HDL cholesterol and triglycerides.

### Alteration of erythrocyte levels of omega-3 and omega-6 PUFAs in diabetic subjects with or without retinopathy

**Figure 1:**
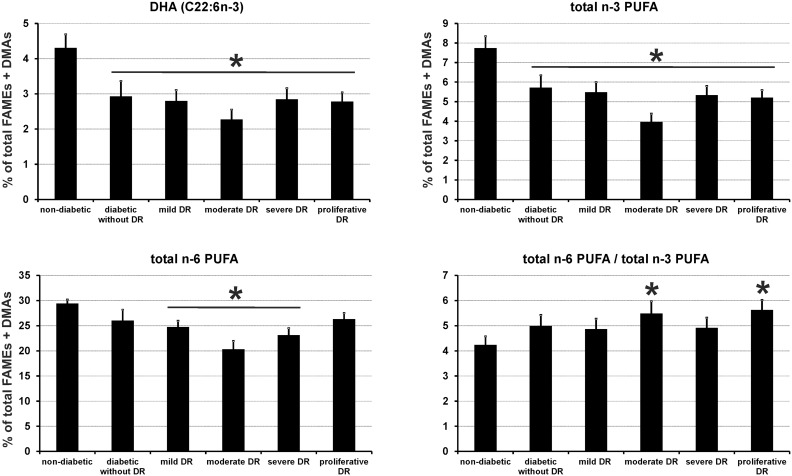
Red blood cell membrane levels of docosahexaenoic acid (DHA), total n-3 PUFA, n-6 PUFA, and total n-6 PUFA/total n-3 PUFA in control subjects and diabetic patients with or without DR. Results are expressed as means ± SEM. * based on Kruskall-Wallis test (*P*<0.05).

The complete fatty acid composition of erythrocytes from control subjects and diabetic patients is presented in **Table 2**. The most striking difference between controls and diabetic patients with or without retinopathy was an alteration of the relative amounts of docosahexaenoic acid (DHA, C22∶6n-3, **Figure 1**), and arachidonic acid (AA, C20∶4n-6). These changes were balanced by a relative increase in saturated fatty acids (C16∶0, C18∶0, and C24∶0) and monounsaturated fatty acids (C18∶1n-9 and C24∶1n-9,). The modification of erythrocyte concentrations of DHA and AA had consequences on the amounts of total n-3 PUFAs which was significantly lowered in all patient groups (*P*<0.05,), as well as on total n-6 PUFAs that were decreased in patients with mild, moderate and severe DR (*P*<0.05) when compared to controls. The variations observed in total PUFAs had a minor impact on the n-6 PUFA to n-3 PUFA ratio.

**Table 2:** Erythrocyte fatty acid composition of controls and diabetic patients without or with diabetic retinopathy at mild, moderate, severe, and proliferative stages (% of total fatty acid methyl esters (FAMEs) + dimethylacetals (DMAs)).

					Patients with diabetic retinopathy							
	Controls		Diabetic patients		mild		moderate		severe		proliferative	
	n = 18		n = 14		n = 12		n = 12		n = 22		n = 24	
	median	*[IQR]*	median	*[IQR]*	median	*[IQR]*	median	*[IQR]*	median	*[IQR]*	median	*[IQR]*
**C14∶0**	**0.43**	*[0.32–0.68]*	**0.48**	*[0.38–0.62]*	**0.73**	*[0.48–1.40]*	**0.64**	*[0.50–1.07]*	**0.85**	*[0.46–1.78]*	**0.48**	*[0.29–0.59]*
**C15∶0**	**0.17**	*[0.15–0.18]*	**0.22**	*[0.16–0.28]*	**0.19**	*[0.16–0.23]*	**0.22**	*[0.19–0.25]*	**0.22**	*[0.18–0.25]*	**0.23**	*[0.18–0.35]*
**DMA16∶0**	**1.85**	*[1.56–1.95]*	**1.77**	*[1.47–2.01]*	**1.65**	*[1.29–2.04]*	**1.50**	*[1.42–1.97]*	**1.33**	*[1.08–1.95]*	**1.74**	*[1.61–2.01]*
**C16∶0**	**20.56**	*[19.89–21.84]*	**21.98**	*[20.07–23.21]*	**23.63**	*[22.16–25.12]*	**24.30** a	*[22.70–26.43]*	**23.75** a	*[22.31–28.35]*	**22.86**	*[20.93–23.69]*
**C16∶1n-9**	**0.12**	*[0.10–0.22]*	**0.20**	*[0.17–0.24]*	**0.17**	*[0.12–0.20]*	**0.19**	*[0.13–0.29]*	**0.17**	*[0.12–0.20]*	**0.15**	*[0.12–0.26]*
**C16∶1n-7**	**0.61**	*[0.50–0.95]*	**0.83**	*[0.57–0.94]*	**0.81**	*[0.64–1.30]*	**0.61**	*[0.54–0.74]*	**0.57**	*[0.45–0.67]*	**0.74**	*[0.40–0.98]*
**C17∶0**	**0.38**	*[0.35–0.41]*	**0.44**	*[0.32–0.47]*	**0.35**	*[0.30–0.46]*	**0.45**	*[0.36–0.57]*	**0.48**	*[0.38–0.56]*	**0.43**	*[0.33–0.51]*
**DMA18∶0**	**3.16**	*[2.36–3.53]*	**3.08**	*[2.44–3.47]*	**2.30**	*[2.06–3.12]*	**2.50**	*[1.94–3.31]*	**2.26** a	*[1.38–3.07]*	**3.13**	*[2.60–3.36]*
**DMA18∶1n-9**	**0.66**	*[0.54–0.70]*	**0.64**	*[0.55–0.72]*	**0.56**	*[0.48–0.65]*	**0.58**	*[0.49–0.76]*	**0.53**	*[0.33–0.68]*	**0.61**	*[0.49–0.68]*
**DMA18∶1n-7**	**0.15**	*[0.12–0.17]*	**0.15**	*[0.12–0.17]*	**0.12**	*[0.09–0.13]*	**0.16**	*[0.12–0.19]*	**0.13**	*[0.08–0.17]*	**0.16**	*[0.12–0.18]*
**C18∶0**	**14.06**	*[13.73–14.55]*	**14.64**	*[13.78–15.64]*	**14.13**	*[12.73–15.09]*	**16.31** a	*[14.78–16.70]*	**14.91** a	*[14.38–16.26]*	**15.10**	*[13.26–15.93]*
**C18∶1trans**	**0.22**	*[0.18–0.26]*	**0.20**	*[0.11–0.24]*	**0.23**	*[0.14–0.35]*	**0.31**	*[0.19–0.46]*	**0.39** a	*[0.15–0.65]*	**0.24**	*[0.15–0.34]*
**C18∶1n-9**	**15.16**	*[14.03–16.30]*	**16.91** a	*[15.50–20.20]*	**17.32**	*[14.45–20.19]*	**16.00**	*[13.65–17.73]*	**15.51**	*[14.07–16.73]*	**15.93**	*[15.26–18.11]*
**C18∶1n-7**	**1.19**	*[1.13–1.36]*	**1.45**	*[1.25–1.66]*	**1.24**	*[1.02–1.61]*	**1.40**	*[1.28–1.56]*	**1.24**	*[1.12–1.34]*	**1.29**	*[1.15–1.44]*
**C18∶2n-6**	**12.87**	*[10.87–13.51]*	**10.58**	*[9.66–14.27]*	**11.30**	*[10.37–14.56]*	**10.15** a	*[8.99–10.86]*	**10.61** a	*[8.79–11.50]*	**11.27**	*[8.74–12.84]*
**C20∶0**	**0.15**	*[0.14–0.17]*	**0.18**	*[0.15–0.20]*	**0.21**	*[0.17–0.23]*	**0.26**	*[0.18–0.32]*	**0.32**	*[0.18–0.34]*	**0.22**	*[0.16–0.31]*
**C18∶3n-6**	**0.09**	*[0.06–0.14]*	**0.12**	*[0.08–0.15]*	**0.12**	*[0.09–0.13]*	**0.09**	*[0.07–0.14]*	**0.10**	*[0.07–0.13]*	**0.12**	*[0.08–0.20]*
**C20∶1n-9**	**0.21**	*[0.19–0.22]*	**0.26**	*[0.21–0.29]*	**0.23**	*[0.21–0.26]*	**0.26**	*[0.23–0.28]*	**0.24**	*[0.20–0.26]*	**0.27**	*[0.23–0.33]*
**C18∶3n-3**	**0.22**	*[0.18–0.28]*	**0.21**	*[0.19–0.30]*	**0.27**	*[0.22–0.28]*	**0.23**	*[0.16–0.30]*	**0.22**	*[0.14–0.27]*	**0.22**	*[0.16–0.25]*
**C20∶2n-6**	**0.20**	*[0.18–0.21]*	**0.23**	*[0.21–0.25]*	**0.25**	*[0.21–0.35]*	**0.20**	*[0.18–0.33]*	**0.28**	*[0.20–0.38]*	**0.23**	*[0.19–0.28]*
**C20∶3n-9**	**0.09**	*[0.07–0.11]*	**0.10**	*[0.08–0.16]*	**0.10**	*[0.07–0.13]*	**0.12**	*[0.08–0.16]*	**0.11**	*[0.08–0.14]*	**0.11**	*[0.09–0.15]*
**C22∶0**	**0.33**	*[0.29–0.42]*	**0.42**	*[0.25–0.44]*	**0.46**	*[0.37–0.64]*	**0.76** a	*[0.46–0.87]*	**0.78** a	*[0.45–1.14]*	**0.62** a	*[0.37–0.86]*
**C20∶3n-9**	**0.10**	*[0.08–0.11]*	**0.10**	*[0.07–0.11]*	**0.08**	*[0.05–0.10]*	**0.08**	*[0.04–0.10]*	**0.05**	*[0.04–0.10]*	**0.10**	*[0.08–0.12]*
**C20∶3n-6**	**1.30**	*[1.22–1.50]*	**1.50**	*[1.12–1.79]*	**1.31**	*[1.01–1.62]*	**1.26**	*[1.07–1.31]*	**1.27**	*[0.95–1.53]*	**1.32**	*[1.13–1.59]*
**C22∶1n-9**	**0.06**	*[0.05–0.06]*	**0.09**	*[0.05–0.14]*	**0.09**	*[0.06–0.14]*	**0.08**	*[0.06–0.10]*	**0.11**	*[0.08–0.17]*	**0.12**	*[0.06–0.18]*
**C20∶4n-6**	**13.04**	*[11.02–14.24]*	**11.33** a	*[7.00–12.79]*	**9.33**	*[8.91–11.95]*	**9.93** a	*[8.41–11.65]*	**9.90** a	*[5.68–11.38]*	**11.68**	*[10.28–13.40]*
**C20∶5n-3**	**0.97**	*[0.71–1.56]*	**1.03**	*[0.90–1.08]*	**0.93**	*[0.61–1.04]*	**0.70** a	*[0.54–0.98]*	**0.94**	*[0.68–1.14]*	**0.65**	*[0.48–0.79]*
**C24∶0**	**0.74**	*[0.63–0.83]*	**0.78**	*[0.65–0.87]*	**0.94**	*[0.81–1.70]*	**1.75** a	*[0.83–2.15]*	**1.66** a	*[0.89–2.31]*	**1.28** a	*[0.62–2.11]*
**C24∶1n-9**	**0.69**	*[0.60–0.83]*	**1.15**	*[0.99–1.47]*	**1.31** a	*[1.14–1.93]*	**2.16** a	*[1.00–2.64]*	**2.00** a	*[1.41–2.55]*	**1.52** a	*[0.90–2.13]*
**C22∶4n-6**	**2.00**	*[1.70–2.18]*	**2.01**	*[0.75–2.21]*	**1.82**	*[1.56–2.19]*	**1.69**	*[1.13–2.31]*	**1.79**	*[1.10–2.19]*	**2.03**	*[1.66–2.63]*
**C22∶5n-6**	**0.35**	*[0.29–0.46]*	**0.30**	*[0.18–0.48]*	**0.34**	*[0.26–0.35]*	**0.36**	*[0.20–0.43]*	**0.35**	*[0.24–0.37]*	**0.37**	*[0.35–0.46]*
**C22∶5n-3**	**2.23**	*[1.43–2.46]*	**1.56** a	*[0.79–2.20]*	**1.67** a	*[1.24–1.81]*	**1.15** a	*[0.99–1.49]*	**1.49** a	*[0.69–1.81]*	**1.59** a	*[1.36–1.66]*
**C22∶6n-3**	**4.51**	*[2.93–5.60]*	**2.85** a	*[2.04–4.48]*	**2.67** a	*[2.32–3.23]*	**2.41** a	*[1.90–2.97]*	**2.75** a	*[1.35–4.23]*	**3.27** a	*[1.87–3.80]*
**total n-3**	**8.33**	*[5.28–9.36]*	**5.99** a	*[4.17–7.33]*	**5.43** a	*[4.38–6.52]*	**3.81** a	*[2.61–5.08]*	**5.68** a	*[2.71–7.42]*	**5.75** a	*[4.17–6.61]*
**total n-6**	**29.92**	*[28.32–31.21]*	**27.92**	*[24.79–30.49]*	**24.94** a	*[21.36–27.31]*	**21.40** a	*[14.38–25.06]*	**24.86** a	*[17.42–28.10]*	**26.75**	*[24.30–30.91]*
**n-6/n-3 ratio**	**3.76**	*[3.23–5.04]*	**4.39**	*[3.79–5.58]*	**4.31**	*[4.07–4.88]*	**4.83** a	*[4.78–5.68]*	**4.75**	*[3.53–5.73]*	**5.08** a	*[4.37–7.02]*
**total DMAs**	**5.80**	*[4.70–6.56]*	**5.90**	*[4.70–6.31]*	**4.34**	*[4.08–5.85]*	**4.78**	*[3.98–6.15]*	**4.36**	*[2.77–6.02]*	**5.77**	*[5.16–6.19]*

a Based on Kruskall-Wallis test, significantly different when compared to controls (*P*<0.05).

### No involvement of plasmenyl-cholines and plasmenyl-ethanolamines in the alteration of erythrocyte levels of PUFAs in patients with DR

As fatty acids can be esterified on several subtypes of phospholipids in cell membranes, we have investigated the origin of the loss of erythrocyte DHA and AA by performing quantitative analyses of individual phospholipid species having phospho-choline (PC and PlsC) and phospho-ethanolamine (PE and PlsE) headgroups. As shown in **Table 3** and **Table 4**, the cause of the lower levels of DHA and AA in erythrocytes was not related to a specific loss of individual species of PlsC and PlsE esterified with these PUFA, as their concentrations were not or only slightly modified.

**Table 3:** Concentration of individual species of phosphatidyl-choline (PC) and plasmenyl-choline (PlsC) in erythrocytes from controls and diabetic patients without or with mild, moderate, severe or proliferative diabetic retinopathy (results are expressed as µg of mg phospholipids).

						Patients with diabetic retinopathy							
		Controls		Diabetic patients		mild		moderate		severe		proliferative	
		n = 18		n = 14		n = 12		n = 12		n = 22		n = 24	
	*[M+H]^+^*	median	*[IQR]*	median	*[IQR]*	median	*[IQR]*	median	*[IQR]*	median	*[IQR]*	median	*[IQR]*
**PC14∶0/16∶0**	706.50	**1.28**	*[1.12–1.58]*	**1.50**	*[1.31–1.78]*	**0.96**	*[0.73–1.17]*	**1.23**	*[1.00–1.56]*	**1.77**	*[1.25–2.36]*	**1.19**	*[0.82–1.59]*
**PlsC16∶0/16∶1**	720.00	**1.41**	*[1.27–1.53]*	**1.31**	*[1.13–1.62]*	**1.03**	*[0.77–1.23]*	**1.36**	*[1.32–1.53]*	**1.29**	*[1.20–2.34]*	**1.09**	*[1.04–1.42]*
**PC16∶0/16∶1**	732.55	**2.52**	*[2.28–3.01]*	**3.73a**	*[3.23–5.67]*	**3.50**	*[2.17–4.54]*	**2.50**	*[2.15–3.38]*	**3.72**	*[3.28–4.05]*	**2.43**	*[1.75–3.63]*
**PC16∶0/16∶0**	734.56	**9.48**	*[8.78–11.59]*	**13.28** a	*[10.60–15.04]*	**9.91**	*[8.26–12.70]*	**10.88**	*[9.51–11.41]*	**12.69** a	*[12.42–19.79]*	**10.97**	*[9.60–12.36]*
**PlsC16∶0/18∶1**	744.58	**1.22**	*[1.01–1.37]*	**1.24**	*[0.92–1.46]*	**0.69**	*[0.57–0.987]*	**0.84**	*[0.70–1.03]*	**0.86**	*[0.83–1.54]*	**0.85**	*[0.76–1.20]*
**PlsC16∶0/18∶0**	746.60	**2.73**	*[2.54–3.00]*	**3.23**	*[2.37–3.85]*	**2.27**	*[1.93–2.40]*	**2.77**	*[2.46–2.92]*	**2.73**	*[2.70–4.11]*	**2.37**	*[2.24–2.82]*
**PC16∶0/18∶2**	758.56	**53.18**	*[50.79–63.76]*	**69.91** a	*[58.99–90.62]*	**54.36**	*[37.55–67.90]*	**54.78**	*[44.27–61.22]*	**61.48** a	*[51.69–77.40]*	**43.31**	*[37.52–68.41]*
**PC16∶0/18∶1**	760.58	**54.50**	*[43.86–58.85]*	**77.70** a	*[64.61–94.58]*	**64.66**	*[53.02–73.96]*	**56.14**	*[45.26–69.14]*	**70.15**	*[66.87–98.07]*	**59.33** a	*[50.02–71.50]*
**PC16∶0/18∶0**	762.59	**3.94**	*[3.26–4.62]*	**4.55**	*[3.51–5.58]*	**3.12**	*[2.06–4.27]*	**3.89**	*[3.64–4.20]*	**5.03**	*[4.11–5.66]*	**3.71**	*[3.41–4.03]*
**PlsC16∶0/20∶3**	768.58	**2.58**	*[2.26–2.82]*	**1.58**	*[1.38–3.25]*	**1.28** a	*[0.77–1.42]*	**2.09**	*[2.00–2.44]*	**1.68**	*[1.24–2.63]*	**1.25**	*[0.93–2.62]*
**PlsC18∶1/18∶0**	772.61	**3.07**	*[2.79–3.32]*	**4.39** a	*[3.34–6.35]*	**2.97**	*[2.62–3.47]*	**3.34**	*[2.75–4.28]*	**3.52**	*[3.31–5.69]*	**3.00**	*[2.79–4.32]*
**PlsC18∶0/18∶0**	774.63	**2.73**	*[2.37–2.87]*	**3.76** a	*[3.31–5.78]*	**2.86**	*[2.41–3.29]*	**3.07**	*[2.61–3.79]*	**4.11** a	*[2.96–6.54]*	**3.24** a	*[2.62–4.83]*
**PC14∶0/22∶5**	780.50	**3.37**	*[2.35–4.58]*	**1.79**	*[1.31–3.88]*	**1.75**	*[1.50–2.91]*	**1.76**	*[1.34–3.48]*	**1.72** a	*[1.40–1.86]*	**1.64** a	*[0.82–2.21]*
**PC16∶0/20∶4**	782.56	**20.16**	*[18.40–22.69]*	**21.55**	*[12.33–43.26]*	**18.90**	*[11.96–23.64]*	**21.66**	*[19.28–23.62]*	**20.05**	*[15.79–30.96]*	**18.36**	*[11.21–24.76]*
**PC18∶1/18∶2+ PC16∶0/20∶3**	784.58	**14.04**	*[12.23–16.91]*	**18.08** a	*[11.48–26.54]*	**12.29**	*[10.12–16.324]*	**15.41**	*[12.18–20.72]*	**15.52**	*[13.29–20.17]*	**10.53**	*[10.00–19.33]*
**PC18∶1/18∶1+ PC18∶0/18∶2**	786.59	**30.26**	*[26.74–34.77]*	**36.19** a	*[28.65–50.82]*	**26.79**	*[22.11–37.17]*	**31.33**	*[23.41–36.60]*	**32.97**	*[24.89–34.68]*	**23.26**	*[19.30–34.33]*
**PC18∶0/18∶1**	788.61	**14.36**	*[13.40–16.04]*	**20.25** a	*[14.89–26.07]*	**13.78**	*[10.16–16.65]*	**14.65**	*[13.52–16.54]*	**15.93**	*[14.48–19.32]*	**14.91**	*[13.33–17.68]*
**PlsC16∶0/22∶6**	790.57	**0.86**	*[0.68–0.98]*	**1.26** a	*[0.48–2.75]*	**0.69**	*[0.50–1.31]*	**1.06**	*[0.88–1.37]*	**0.99**	*[0.68–1.86]*	**0.84**	*[0.74–1.70]*
**PlsC18∶0/20∶4+ PlsC16∶0/22∶4**	794.60	**2.30**	*[1.98–2.43]*	**1.29**	*[0.89–2.44]*	**1.20** a	*[0.84–1.57]*	**1.70**	*[1.52–2.25]*	**1.50** a	*[1.17–2.11]*	**1.04** a	*[1.02–2.07]*
**PlsC18∶1/20∶2**	796.61	**2.82**	*[2.32–3.20]*	**2.28**	*[1.39–2.94]*	**1.61**	*[1.19–1.86]*	**2.49**	*[2.03–3.49]*	**1.79**	*[1.50–2.90]*	**1.40**	*[1.08–2.72]*
**PC18∶2/20∶4+ PC16∶0/22∶6**	806.56	**8.84**	*[7.75–11.10]*	**8.04**	*[3.94–13.25]*	**5.49**	*[4.22–7.50]*	**7.48**	*[4.47–9.81]*	**6.41**	*[5.22–7.44]*	**5.12**	*[2.95–7.03]*
**PC18∶1/20∶4+ PC16∶0/22∶5**	808.58	**6.08**	*[5.27–7.99]*	**5.12**	*[2.61–11.88]*	**3.28**	*[3.06–4.72]*	**4.92**	*[3.70–6.30]*	**4.47**	*[3.45–5.30]*	**4.17**	*[2.28–5.96]*
**PC18∶0/20∶4**	810.59	**10.62**	*[9.56–11.53]*	**10.69**	*[6.24–22.40]*	**9.15**	*[6.40–12.17]*	**10.18**	*[8.74–12.73]*	**10.93**	*[7.59–11.47]*	**7.48**	*[5.13–14.75]*
**PC18∶0/20∶3**	812.61	**3.22**	*[2.62–4.31]*	**3.98**	*[2.84–4.87]*	**2.95**	*[2.76–3.86]*	**4.43**	*[2.28–5.83]*	**2.74**	*[2.53–3.55]*	**2.54**	*[2.22–4.39]*
**PC18∶0/20∶2**	814.60	**0.90**	*[0.81–0.92]*	**1.33** a	*[0.96–1.92]*	**0.79**	*[0.65–0.94]*	**0.87**	*[0.73–1.20]*	**1.50**	*[1.46–1.63]*	**0.75**	*[0.62–1.00]*
**PlsC18∶1/22∶6**	816.35	**0.92**	*[0.82–1.08]*	**1.53** a	*[1.08–2.14]*	**0.85**	*[0.59–1.29]*	**0.84**	*[0.73–1.04]*	**1.33** a	*[1.30–2.13]*	**0.71**	*[0.68–1.31]*
**PlsC18∶0/22∶6**	818.70	**0.60**	*[0.44–0.67]*	**0.86** a	*[0.64–1.22]*	**0.47**	*[0.32–0.55]*	**0.55**	*[0.45–0.67]*	**0.89** a	*[0.56–1.73]*	**0.38**	*[0.36–0.82]*
**PlsC18∶1/22∶4**	820.55	**0.72**	*[0.61–0.79]*	**0.74** a	*[0.52–1.40]*	**0.52**	*[0.39–0.57]*	**0.58**	*[0.42–0.67]*	**0.66**	*[0.63–0.97]*	**0.54**	*[0.50–0.67]*
**PlsC18∶0/22∶4**	822.55	**0.52**	*[0.44–0.57]*	**0.49**	*[0.36–0.83]*	**0.43**	*[0.41–0.45]*	**0.53**	*[0.33–0.67]*	**0.52**	*[0.39–0.82]*	**0.38**	*[0.34–0.58]*
**PlsC18∶1/22∶2**	824.25	**0.50**	*[0.47–0.56]*	**0.56**	*[0.37–0.79]*	**0.43**	*[0.34–0.61]*	**0.63**	*[0.38–0.67]*	**0.58**	*[0.35–0.80]*	**0.52**	*[0.33–0.71]*
**PlsC18∶1/22∶1**	826.25	**0.25**	*[0.21–0.26]*	**0.52** a	*[0.35–1.29]*	**0.64** a	*[0.38–0.87]*	**0.43** a	*[0.37–0.66]*	**0.53** a	*[0.47–1.31]*	**0.32** a	*[0.24–0.85]*
**PC18∶1/22∶6**	832.58	**0.70**	*[0.57–0.79]*	**0.68** a	*[0.42–1.34]*	**0.51**	*[0.38–0.80]*	**0.59**	*[0.45–0.81]*	**0.79**	*[0.78–1.04]*	**0.54**	*[0.49–0.95]*
**PC18∶0/22∶6**	834.59	**3.07**	*[2.55–3.40]*	**2.45**	*[1.63–3.71]*	**1.89**	*[1.30–2.53]*	**2.51**	*[1.42–3.90]*	**1.88** a	*[1.56–2.49]*	**1.55** a	*[0.98–2.15]*
**PC18∶0/22∶5+ PC18∶1/22∶4**	836.61	**0.98**	*[0.79–1.20]*	**1.09**	*[0.62–2.11]*	**1.10**	*[0.76–1.16]*	**0.81**	*[0.68–1.32]*	**1.26**	*[0.84–1.58]*	**0.64**	*[0.49–1.02]*
**PC18∶0/22∶4**	838.62	**0.41**	*[0.32–0.53]*	**0.61**	*[0.50–0.82]*	**0.29**	*[0.24–0.39]*	**0.41**	*[0.34–0.56]*	**0.54**	*[0.52–0.76]*	**0.30**	*[0.24–0.66]*
**PC20∶6/22∶6**	850.50	**0.20**	*[0.15–0.23]*	**0.40** a	*[0.21–0.76]*	**0.23**	*[0.16–0.30]*	**0.24**	*[0.20–0.27]*	**0.32**	*[0.27–0.45]*	**0.23**	*[0.19–0.38]*
**PC20∶3/22∶6+ PC20∶4/22∶5+ PC20∶5/22∶4**	856.60	**0.21**	*[0.17–0.27]*	**0.35** a	*[0.17–0.75]*	**0.18**	*[0.12–0.23]*	**0.25**	*[0.17–0.36]*	**0.35**	*[0.34–0.59]*	**0.22**	*[0.20–0.50]*
**PC22∶6/22∶6**	878.56	**0.16**	*[0.12–0.20]*	**0.25** a	*[0.08–0.44]*	**0.15**	*[0.10–0.19]*	**0.25**	*[0.12–0.31]*	**0.24**	*[0.15–0.31]*	**0.18**	*[0.13–0.29]*
**Total PlsC**	**-**	**22.06**	*[19.22–23.94]*	**27.41** a	*[20.61–35.60]*	**17.89**	*[16.21–19.47]*	**22.64**	*[19.98–24.64]*	**24.45**	*[21.79–33.44]*	**21.01**	*[19.24–24.05]*
**Total PlsC with 22∶6**	**-**	**2.34**	*[2.13–2.62]*	**3.77** a	*[2.36–5.19]*	**2.09**	*[1.74–2.92]*	**2.49**	*[2.14–3.00]*	**3.24** a	*[3.20–5.66]*	**1.99**	*[1.60–3.91]*
**Total PC with 22∶6**	**-**	**12.84**	*[10.99–16.55]*	**11.94**	*[9.00–17.88]*	**8.98**	*[6.37–11.48]*	**11.30**	*[7.04–15.37]*	**10.22**	*[8.32–10.74]*	**8.31**	*[5.47–10.98]*
**Total PlsC + PC with 22∶6**	**-**	**15.13**	*[13.60–18.98]*	**16.90** a	*[13.27–23.45]*	**11.45**	*[8.636–13.96]*	**13.04**	*[9.34–18.43]*	**13.14**	*[12.80–17.48]*	**10.30**	*[9.01–14.08]*
**Total PlsC + PC**	**-**	**272.68**	*[240.85–299.96]*	**335.43** a	*[280.23–460.04]*	**261.47**	*[198.72–315.45]*	**259.54**	*[227.87–300.87]*	**292.86**	*[270.20–324.37]*	**224.70**	*[209.82–331.66]*

Abbreviations of individual PC and PlsC species are as follows: position on the glycerol backbone as shown as sn-1/sn-2 of the fatty acid and fatty alcohol radicals (abbreviated as number of carbons: number of double bonds).

a Based on Kruskall-Wallis test, significantly different when compared to controls (*P*<0.05).

**Table 4:** Concentration of individual species of phosphatidyl-ethanolamine (PE) and plasmenyl- ethanolamine (PlsE) in erythrocytes from controls and diabetic patients without or with mild, moderate, severe or proliferative diabetic retinopathy (results are expressed as µg of mg phospholipids for PE species and as ratio to internal standard PC14∶0/14∶0 for PlsE species).

						Patients with diabetic retinopathy							
		Controls		Diabetic patients		mild		moderate		severe		proliferative	
		n = 18		n = 14		n = 12		n = 12		n = 22		n = 24	
	*[M+H]+ or MS/MS transition*	median	*[IQR]*	median	*[IQR]*	median	*[IQR]*	median	*[IQR]*	median	*[IQR]*	median	*[IQR]*
**PE14∶1/16∶2 b**	658.40	*<0.01*	*-*	*<0.01*	*-*	*<0.01*	*-*	**0.01**	*[0.00–0.02]*	*<0.01*	*-*	*<0.01*	*-*
**PE16∶0/16∶1**	690.53	**0.53**	*[0.45–0.80]*	**0.43**	*[0.23–0.74]*	**0.58**	*[0.42–0.63]*	**0.36**	*[0.30–0.43]*	**0.46**	*[0.31–0.56]*	**0.34**	*[0.25–0.49]*
**PE16∶0/16∶0**	692.54	**1.12**	*[0.76–1.29]*	**0.89**	*[0.40–1.50]*	**0.93**	*[0.76–1.06]*	**0.64**	*[0.39–0.83]*	**0.93**	*[0.60–1.28]*	**0.63**	*[0.44–0.93]*
**PE16∶1/18∶2**	714.50	**0.69**	*[0.50–0.81]*	**0.68**	*[0.48–1.09]*	**1.15**	*[0.77–1.21]*	**0.59**	*[0.50–0.68]*	**0.71**	*[0.61–0.77]*	**0.54**	*[0.30–0.69]*
**PE16∶0/18∶2+ PE16∶1/18∶1**	716.54	**10.86**	*[9.35–14.48]*	**11.97**	*[5.88–16.70]*	**14.22**	*[13.42–15.89]*	**8.34** a	*[7.62–8.76]*	**11.96**	*[7.56–13.30]*	**8.53**	*[5.95–11.28]*
**PE16∶0/18∶1**	718.56	**37.91**	*[30.40–47.15]*	**41.33**	*[34.13–47.58]*	**54.62** a	*[41.54–56.27]*	**32.60**	*[28.65–35.12]*	**41.68**	*[33.18–50.10]*	**31.26**	*[23.09–39.71]*
**PE16∶0/18∶0**	720.57	*<0.01*	*-*	*<0.01*	*-*	*<0.01*	*-*	**0.02**	*[0.00–0.18]*	*<0.01*	*-*	*<0.01*	*-*
**PE16∶1/20∶4+ PE16∶0/20∶5**	738.50	*<0.01*	*-*	*<0.01*	*-*	*<0.01*	*-*	*<0.01*	*-*	*<0.01*	*-*	**0.20**	*[0.00–0.68]*
**PE16∶0/20∶4+ PE18∶2/18∶2**	740.54	**14.75**	*[9.45–16.65]*	**13.65**	*[7.91–17.77]*	**13.73**	*[12.96–18.52]*	**9.18**	*[0.74–16.47]*	**15.32**	*[10.10–18.39]*	**14.23**	*[6.62–16.35]*
**PE16∶0/20∶3+ PE18∶1/18∶2**	742.56	**10.17**	*[7.92–13.96]*	**9.92**	*[6.17–12.14]*	**12.02**	*[10.97–14.93]*	**8.04**	*[5.50–11.05]*	**11.19**	*[7.18–11.49]*	**8.84**	*[4.67–9.13]*
**PE18∶1/18∶1+ PE18∶0/18∶2**	744.57	**14.19**	*[11.39–18.07]*	**15.09**	*[12.19–18.87]*	**18.55**	*[16.70–19.93]*	**13.94**	*[10.30–14.96]*	**14.72**	*[13.39–15.35]*	**12.20**	*[8.65–15.54]*
**PE18∶0/18∶1**	746.59	**10.78**	*[8.17–11.75]*	**10.42**	*[8.94–12.26]*	**12.28**	*[8.90–14.75]*	**8.53**	*[7.03–8.80]*	**12.65**	*[10.56–13.04]*	**8.54**	*[6.86–10.72]*
**PE18∶0/18∶0**	748.55	*<0.01*	*-*	*<0.01*	*-*	*<0.01*	*-*	**0.13**	*[0.00–0.30]*	*<0.01*	*-*	*<0.01*	*-*
**PE18∶2/20∶4+ PE16∶0/22∶6**	765.54	**11.61**	*[9.02–14.88]*	**8.23** a	*[5.00–12.09]*	**7.67** a	*[5.95–11.06]*	**4.28**	*[4.01–14.09]*	**10.33**	*[5.67–11.45]*	**8.31** a	*[1.63–11.25]*
**PE18∶1/20∶4+ PE16∶0/22∶5+ PE18∶0/20∶5**	766.56	**16.08**	*[14.05–18.74]*	**11.87** a	*[5.71–14.33]*	**10.87**	*[9.40–17.69]*	**13.42**	*[9.22–16.08]*	**13.24**	*[7.77–13.74]*	**12.54**	*[3.27–15.08]*
**PE18∶0/20∶4**	768.57	**20.97**	*[18.64–25.50]*	**16.22** a	*[10.34–21.32]*	**14.99**	*[12.724–22.21]*	**13.87**	*[11.34–21.73]*	**18.42**	*[10.64–21.98]*	**18.78**	*[6.52–20.95]*
**PE18∶0/20∶3**	770.59	**0.56**	*[0.15–1.58]*	**1.04**	*[0.64–1.30]*	**1.10**	*[0.36–1.51]*	**0.77**	*[0.17–1.21]*	**1.26**	*[0.74–1.43]*	**0.59**	*[0.39–0.98]*
**PE18∶0/20∶2**	772.60	**0.48**	*[0.30–0.69]*	**0.73**	*[0.61–1.07]*	**0.72**	*[0.21–1.10]*	**0.31**	*[0.19–0.82]*	**0.69**	*[0.57–0.90]*	**0.49**	*[0.36–0.64]*
**PE18∶0/20∶1**	774.56	**0.23**	*[0.15–0.40]*	**0.37**	*[0.08–0.41]*	**0.23**	*[0.06–0.37]*	**0.30**	*[0.24–0.69]*	**0.20**	*[0.01–0.50]*	**0.20**	*[0.15–0.29]*
**PE18∶0/20∶0**	776.50	*<0.01*	*-*	*<0.01*	*-*	*<0.01*	*-*	*<0.01*	*-*	*<0.01*	*-*	**0.04**	*[0.00–0.11]*
**PE20∶4/20∶4**	788.54	**1.72**	*[1.37–2.02]*	**1.34**	*[0.57–2.03]*	**1.43**	*[1.11–1.93]*	**1.15**	*[0.96–1.25]*	**1.56**	*[0.74–2.01]*	**0.91**	*[0.52–1.39]*
**PE18∶1/22∶6**	790.56	**6.86**	*[5.99–7.57]*	**4.57**	*[3.14–6.34]*	**4.93**	*[3.08–6.11]*	**3.14** a	*[1.21–4.63]*	**5.46**	*[1.79–7.21]*	**3.25** a	*[1.41–4.92]*
**PE18∶0/22∶6**	792.57	**6.64**	*[6.31–7.41]*	**4.71**	*[2.62–7.11]*	**3.51** a	*[2.98–5.55]*	**4.44** a	*[3.25–5.24]*	**5.18** a	*[1.76–5.70]*	**3.60** a	*[1.12–5.29]*
**PE18∶0/22∶5+ PE18∶1/22∶4**	794.59	**5.89**	*[5.08–6.26]*	**3.69**	*[2.39–6.75]*	**3.49**	*[3.02–6.30]*	**4.09**	*[1.60–5.01]*	**5.10**	*[1.91–5.57]*	**3.82**	*[1.75–5.33]*
**PE18∶0/22∶4+ PE20∶0/20∶4**	796.60	**2.94**	*[2.70–3.24]*	**2.53**	*[1.30–3.07]*	**2.61**	*[1.77–3.52]*	**3.01**	*[1.93–3.39]*	**3.76**	*[1.21–4.01]*	**1.64**	*[1.07–3.91]*
**PE20∶0/20∶3+ PE18∶2/22∶1**	798.62	**0.04**	*[0.00–0.12]*	**0.03**	*[0.00–0.37]*	**0.39** a	*[0.16–0.45]*	*<0.01*	*-*	**0.05**	*[0.00–0.39]*	**0.12** a	*[0.00–0.23]*
**PE20∶0/20∶2**	800.65	*<0.01*	*-*	*<0.01*	*-*	*<0.01*	*-*	**0.05**	*[0.00–0.12]*	*<0.01*	*-*	*<0.01*	*-*
**PE20∶4/22∶6**	812.52	**0.75**	*[0.51–0.91]*	**0.45**	*[0.21–0.84]*	**0.31** a	*[0.23–0.46]*	**0.40** a	*[0.23–0.46]*	**0.32** a	*[0.18–0.48]*	**0.40** a	*[0.17–0.52]*
**PE20∶4/22∶5+ PE20∶3/22∶6**	814.56	**0.47**	*[0.33–0.59]*	**0.34**	*[0.17–0.54]*	**0.26** a	*[0.12–0.29]*	**0.12** a	*[0.01–0.36]*	**0.21** a	*[0.13–0.59]*	**0.13** a	*[0.07–0.34]*
**PE20∶2/22∶6+ PE20∶4/22∶4**	816.50	*<0.01*	*-*	*<0.01*	*-*	*<0.01*	*-*	**0.11**	*[0.00–0.24]*	*<0.01*	*-*	**0.01**	*[0.00–0.11]*
**PE20∶1/22∶6**	818.56	**0.63**	*[0.33–0.81]*	**0.27**	*[0.06–0.72]*	**0.30**	*[0.25–0.47]*	**0.35**	*[0.24–0.55]*	**0.28**	*[0.18–0.40]*	**0.20**	*[0.08–0.38]*
**PE20∶0/22∶6+ PE20∶2/22∶4**	820.55	*<0.01*	*-*	*<0.01*	*-*	*<0.01*	*-*	*<0.01*	*-*	*<0.01*	*-*	**0.01** a	*[0.00–0.14]*
**PE20∶0/22∶4**	824.63	**0.15**	*[0.09–0.29]*	**0.10**	*[0.06–0.26]*	**0.16**	*[0.02–0.25]*	**0.15**	*[0.06–0.24]*	**0.10**	*[0.00–0.25]*	**0.06**	*[0.03–0.16]*
**PE22∶6/22∶6**	836.56	*<0.01*	*-*	*<0.01*	*-*	*<0.01*	*-*	*<0.01*	*-*	*<0.01*	*-*	*<0.01*	*-*
**PE22∶5/22∶6**	838.60	*<0.01*	*-*	*<0.01*	*-*	*<0.01*	*-*	*<0.01*	*-*	*<0.01*	*-*	*<0.01*	*-*
**PE22∶4/22∶6**	840.50	*<0.01*	*-*	*<0.01*	*-*	*<0.01*	*-*	*<0.01*	*-*	*<0.01*	*-*	*<0.01*	*-*
**PE22∶3/22∶6**	842.60	*<0.01*	*-*	*<0.01*	*-*	*<0.01*	*-*	*<0.01*	*-*	*<0.01*	*-*	*<0.01*	*-*
**PlsE16∶0/20∶4**	722 ->303	**0.0001**	*[0.0001–0.0001]*	*<0.0001*	*-*	*<0.0001*	*-*	**0.0013** a	*[0.0013–0.0013]*	**0.0001**	*[0.0000–0.0011]*	**0.0003** a	*[0.0000–0.0038]*
**PlsE16∶0/20∶3**	724 ->305	*<0.0001*	*-*	*<0.0001*	*-*	*<0.0001*	*-*	*<0.0001*	*-*	*<0.0001*	*-*	*<0.0001*	*-*
**PlsE18∶0/18∶1**	728 ->281	**0.0074**	*[0.0073–0.0074]*	**0.0026**	*[0.0013–0.0279]*	**0.0022**	*[0.0018–0.0048]*	**0.0199**	*[0.0198–0.0304]*	**0.0050**	*[0.0017–0.0094]*	**0.0115**	*[0.0068–0.0177]*
**PlsE16∶0/22∶6**	746 ->327	**0.0001**	*[0.0001–0.0001]*	*<0.0001*	*-*	*<0.0001*	*-*	**0.0005**	*[0.0000–0.0010]*	*<0.0001*	*-*	*<0.0001*	*-*
**PlsE18∶1/20∶4**	748 ->303	**0.0001**	*[0.0001–0.0001]*	*<0.0001*	*-*	*<0.0001*	*-*	**0.0005** a	*[0.0000–0.0015]*	*<0.0001*	*-*	**0.0001**	*[0.0000–0.0017]*
**PlsE16∶0/22∶5**	748 ->329	**0.0001**	*[0.0001–0.0001]*	*<0.0001*	*-*	*<0.0001*	*-*	**0.0011** a	*[0.0010–0.0011]*	**0.0001**	*[0.0000–0.0010]*	**0.0001** a	*[0.0000–0.0029]*
**PlsE18∶0/20∶4**	750 ->303	**0.0160**	*[0.0159–0.0160]*	**0.0070**	*[0.0043–0.0113]*	**0.0050**	*[0.0036–0.0149]*	**0.0065**	*[0.0064–0.0154]*	**0.0086**	*[0.0057–0.0097]*	**0.0095**	*[0.0010–0.0202]*
**PlsE16∶0/22∶4**	750 ->331	**0.0001**	*[0.0001–0.0001]*	*<0.0001*	*-*	*<0.0001*	*-*	**0.0016**	*[0.0012–0.0015]*	**0.0001**	*[0.0000–0.0014]*	**0.0004**	*[0.0004–0.0035]*
**PlsE18∶1/22∶6**	772 ->327	*<0.0001*	*-*	*<0.0001*	*-*	*<0.0001*	*-*	*<0.0001*	*-*	*<0.0001*	*-*	*<0.0001*	*-*
**PlsE18∶0/22∶6**	774 ->327	**0.0003**	*[0.0002–0.0002]*	*<0.0001*	*-*	*<0.0001*	*-*	**0.0005**	*[0.0000–0.0010]*	*<0.0001*	*-*	**0.0002**	*[0.0000–0.0005]*
**PlsE18∶1/22∶4**	776 ->331	**0.0001**	*[0.0001–0.0001]*	*<0.0001*	*-*	*<0.0001*	*-*	**0.0001**	*[0.0000–0.0007]*	**0.0001**	*[0.0000–0.0001]*	**0.0001**	*[0.0000–0.0008]*
**PlsE18∶0/22∶5**	776 ->329	**0.0026**	*[0.0025–0.0025]*	**0.0012**	*[0.0005–0.0030]*	**0.0008**	*[0.0002–0.0027]*	**0.0008**	*[0.0008–0.0033]*	**0.0015**	*[0.0005–0.0016]*	**0.0020**	*[0.0002–0.0043]*
**PlsE18∶0/22∶4**	778 ->329	**0.0204**	*[0.0203–0.0203]*	**0.0040** a	*[0.0013–0.0074]*	**0.0037** a	*[0.0033–0.0060]*	**0.0076** a	*[0.0005–0.01528]*	**0.0071** a	*[0.0022–0.0099]*	**0.0039** a	*[0.0002–0.0068]*
**total PlsE**	**-**	**0.0473**	*[0.0472–0.0472]*	**0.0233** a	*[0.0111–0.0321]*	**0.0126** a	*[0.0094–0.0374]*	**0.0317**	*[0.0317–0.0663]*	**0.0235** a	*[0.0167–0.0293]*	**0.0394**	*[0.0178–0.0654]*
**total PlsE with 22∶6**	**-**	**0.0004**	*[0.0003–0.0003]*	*<0.0001* a	*-*	*<0.0001* a	*-*	**0.0010**	*[0.0000–0.0020]*	**0.0001** a	*[0.0000–0.0002]*	**0.0002**	*[0.0000–0.0006]*
**total PE with 22∶6**	**-**	**27.35**	*[23.49–32.80]*	**17.53** a	*[11.41–26.08]*	**15.57** a	*[13.17–25.91]*	**13.30** a	*[11.35–25.90]*	**23.34** a	*[10.04–28.40]*	**17.45** a	*[4.62–23.60]*
**total PE**	**-**	**186.75**	*[149.97–206.15]*	**163.49**	*[110.80–206.00]*	**169.34**	*[158.12–217.51]*	**125.12** a	*[113.43–159.51]*	**180.88**	*[125.98–203.57]*	**147.46** a	*[84.46–170.59]*

[M+H]+ for PE species and MS/MS transition for PlsE species.

Abbreviations of individual PE and PlsE species are as follows: position on the glycerol backbone as shown as sn-1/sn-2 of the fatty acid and fatty alcohol radicals (abbreviated as number of carbons: number of double bonds).

a Based on Kruskall-Wallis test, significantly different when compared to controls (*P*<0.05).

### The loss of N-6 and N-3 PUFAs in patients with DR is the consequence of modifications of erythrocyte levels of PE

Except for specific modifications in diabetic subjects without retinopathy, no major change was observed in individual species of PC esterified or not to DHA and AA in patients with DR (**Table 3**). Only the concentrations of PC18∶0∶22∶6 were significantly lowered in patients with severe and proliferative DR when compared to controls (*P*<0.05).

**Figure 2:**
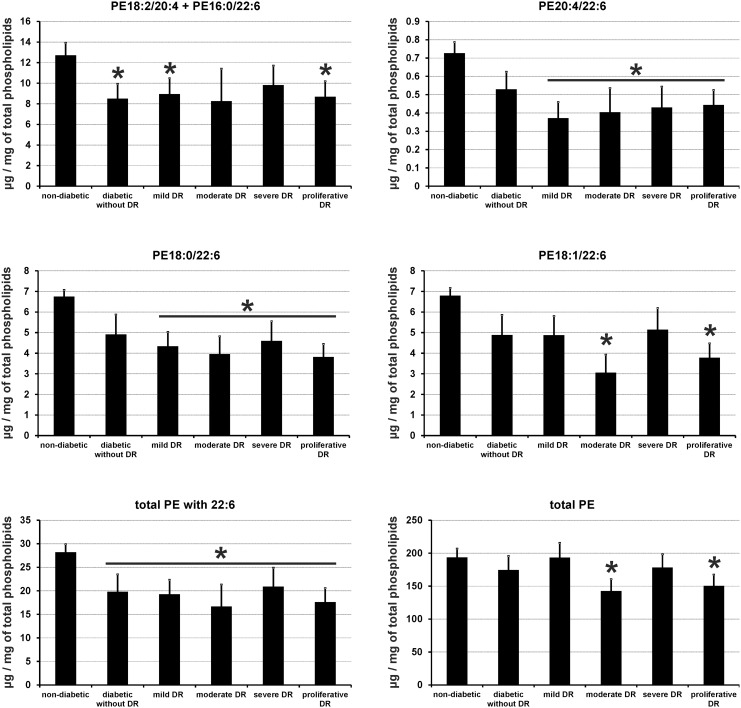
Red blood cell membrane levels of selected phospatidyl- ethanolamine (PE) species esterified with docosahexaenoic acid (22∶6 or DHA,) and/or arachidonic acid (20∶4 or AA), and total PE esterified or not with DHA in control subjects and diabetic patients with or without DR. Results are expressed as means ± SEM. * based on Kruskall-Wallis test (*P*<0.05).

Contrary to PC species, erythrocyte concentrations of individual species of PE were largely modified in all diabetic patients having or not a retinopathy (**Table 4**). These changes were concentrated to PE species esterified either to both AA and DHA (PE20∶4/22∶6), or to DHA or AA only (PE18∶0/22∶6, PE18∶1/22∶6, PE18∶2/20∶4+ PE16∶0/22∶6) (**Figure 2**). The alteration of the concentrations of these PE species lowered the total pool of PE esterified to DHA in all diabetic patients with or without retinopathy when compared to controls (*P*<0.05). However, since these modifications were at least partly balanced by an increase in the concentrations of other PE species (particularly PE16∶0/18∶1), they had no consequence on the amount of total PE except for patients with moderate and proliferative DR.

### Specific alterations in the concentrations of individual species of PC and PlsC in diabetic subjects without retinopathy

Diabetic subjects without retinopathy were characterized by specific modifications in PC and PlsC species that were not observed in controls or in diabetic patients with DR (**Table 3**). These changes consisted in significantly increased levels of PC or PlsC species esterified with saturated and/or monounsaturated fatty acids (namely PC16∶0/16∶1, PC16∶0/16∶0, PC16∶0/18∶1, PC18∶1/18∶1, PC18∶0/18∶1, PlsC18∶1/18∶0, PlsC18∶0/18∶0, and PlsC18∶1/22∶1, *P*<0.05) or with PUFAs including AA and DHA (namely PC18∶1/18∶2, PC16∶0/20∶3, PC18∶0/18∶2, PC18∶0/20∶2, PC18∶1/22∶6, PC20∶6/22∶6, PC20∶3/22∶6, PC20∶4/22∶6, PC20∶5/22∶4, PC22∶6/22∶6, PlsC16∶0/22∶6, and PlsC18∶1/22∶4, *P*<0.05). As a consequence, the total amount of phospholipids having a phospho-choline headgroup was significantly increased in erythrocytes from these subjects (median *[IQR]* for total PlsC of 22.06 *[19.22–23.94]* and 27.41 *[20.61–35.60]* µg of mg of phospholipids in control subjects and diabetic patients without retinopathy, respectively, P<0.05; median *[IQR]* for total PC + PlsC of 272.68 *[240.85–299.96]* and 335.43 *[280.23–460.04]* µg of mg of phospholipids in control subjects and diabetic patients without retinopathy, respectively, P<0.05).

## Discussion

Our study pointed out several quantitative changes in erythrocyte lipids in diabetic patients with or without DR. To our knowledge, this kind of lipidomic study following different stages of retinal microvascular complication of diabetes has never been performed before and represents the largest series of diabetic patients in a lipidomic study. As our study included almost exclusively type 2 diabetic patients, our observations are likely to be restricted to this type of diabetes.

Our data has shown a global decrease in the levels of n-3 and n-6 LCPUFAs in erythrocyte membranes in all diabetic patients independently from the stage of retinopathy. This decrease in LC-PUFAs was balanced by increased levels of saturated and monounsaturated fatty acids. The structural analysis of phospholipids by LC-ESI-MS enabled us to determine which phospholipid species were quantitatively affected by these changes. Whereas plasmalogens were not implicated, our results showed reduced concentrations of PE species esterified with DHA and AA in all diabetic patients balanced by an increase in PE esterified with saturated fatty acids. Total levels of PC and PlsC were unaffected in DR, but increased in diabetic patients without retinopathy. The alteration of LCPUFA levels in diabetic patients was not related to PlsC or PlsE.

Our results are in accordance with previous observations on diabetic patients and animal models of diabetes. In diabetic rats, LC-ESI MS analyses on myocardial cells showed a 27%-decrease in the levels of PE esterified with AA and DHA without any modification of PC amounts. However, a 44%-increase in PlsE and phosphatidyl-inositol contents was described [15]. In another study on diabetic rat myocardial cells, a significant decrease in AA and DHA in PC and PE and a higher phospholipase A2 activity were observed [16]. These changes were associated to increased concentrations of PlsE, suspected to be a protective mechanism against insulin metabolism changes. In pregnant women affected by gestational diabetes, a shift from unsaturated fatty acids to saturated and monounsaturated fatty acids was observed in erythrocytes [17]. In addition to confirming comparable alterations in patients with DR, our data show a specific increase in the concentrations of several PC and PlsC species esterified with monounsaturated fatty acids in diabetic patients without retinopathy. We hypothesize that this may be the result of an ultimate compensatory mechanism to balance the loss of AA- and DHA-rich PE species observed in diabetes.

One cause of the reduction of the unsaturation index (switch from unsaturated to more saturated fatty acids) of cell membranes could be related to a decreased activity of delta-5 and delta-6 desaturase enzymes that are responsible for the biosynthesis of LCPUFAs. Indeed, the activity of these enzymes was shown to be modified in diabetes [28].

Such biochemical modifications in red blood cell membranes are suspected to play a key role in the pathophysiology of diabetes as they alter the permeability and the viscoelastic properties of membranes [29], [30]. A direct consequence would be a reduction of erythrocytes deformability, thus promoting microvascular complications of diabetes as cellular deformability is a critical factor modulating blood flow in microcapillaries [31]. This hypothesis was corroborated by two works showing an association between reduced deformability of erythrocytes and microvascular complications in diabetic patients [32], [33]. It was also hypothesized that increased membrane rigidity resulting from a decreased unsaturation index inhibits the integration of insulin-dependent glucose receptors (GLUT4) into plasma membrane, thus reducing glucose effectiveness and resulting in increased insulin secretion. This was confirmed by Borkman et al., who described a correlation between LCPUFA concentrations in membrane phospholipids of skeletal muscle cells and insulin sensitivity [34].

Such modifications in LCPUFAs bioavailability make also attractive the hypothesis of an impact of the reduced unsaturation index on endothelial function. This idea is supported by data from a study on human retinal vascular endothelial cells treated with VEGF and pro-inflammatory cytokines, and showing that a pre-treatment with DHA inhibits cellular inflammation through the NF-κB pathway [35]. Other studies reported the protective effects of n-3 LCPUFAs on other biochemical pathways involved in DR pathophysiology, namely the PKC pathway promoting VEGF release through diacylglycerols [36], the inducible nitric oxide synthase pathway producing free radicals [37], the endothelin-1 pathway processing vasoconstriction [38], and protein glycosylation [39]. On the other hand, another n-3 LCPUFA, eicosapentanoic acid, is known to prevent pericyte degeneration [40]. Finally, Tikhonenko and collaborators have recently reported a preservation of retinal capillaries in diabetic rats supplemented with DHA [19]. This was associated with an enhanced life span and a reduction of retinal inflammatory markers, suggesting potential benefits of n-3 LCPUFA supplementation in the prevention of DR in diabetic patients.

Our study has several limitations which are, i) the absence of serine- and inositol-esterified phospholipids in our analyses, ii) the absence of statistical difference for glycated hemoglobin between patients with or without DR, iii) the absence of evaluation of dietary intake of LCPUFAs. Moreover, the rate of macro-vascular and micro-vascular complications of diabetes of our cohort increased with the progression of diabetic retinopathy without any statistical significance between the groups, probably due to the size of the cohort. Still, these rates are in the range of those reported in the literature and we do not exclude that the modifications observed in red blood cell lipids may also relate these complications, and iv) due to the recruitment of our patients in our series, we cannot draw firm conclusion for type 1 diabetic patients because they were poorly represented. Finally, an increased number of subjects would have sharpened our results, and a long term follow-up would have enabled us to determine the effect of a better control of diabetes on the lipid composition of erythrocyte membranes.
